# Developmental gene expression profiles of the human pathogen *Schistosoma japonicum*

**DOI:** 10.1186/1471-2164-10-128

**Published:** 2009-03-25

**Authors:** Geoffrey N Gobert, Luke Moertel, Paul J Brindley, Donald P McManus

**Affiliations:** 1 Division of Infectious Diseases & Immunology, Queensland Institute of Medical Research, Brisbane, Queensland 4029, Australia; 2 Department of Microbiology, Immunology & Tropical Medicine, George Washington University Medical Center, Washington, DC 20037, USA

## Abstract

**Background:**

The schistosome blood flukes are complex trematodes and cause a chronic parasitic disease of significant public health importance worldwide, schistosomiasis. Their life cycle is characterised by distinct parasitic and free-living phases involving mammalian and snail hosts and freshwater. Microarray analysis was used to profile developmental gene expression in the Asian species, *Schistosoma japonicum*. Total RNAs were isolated from the three distinct environmental phases of the lifecycle – aquatic/snail (eggs, miracidia, sporocysts, cercariae), juvenile (lung schistosomula and paired but pre-egg laying adults) and adult (paired, mature males and egg-producing females, both examined separately). Advanced analyses including ANOVA, principal component analysis, and hierarchal clustering provided a global synopsis of gene expression relationships among the different developmental stages of the schistosome parasite.

**Results:**

Gene expression profiles were linked to the major environmental settings through which the developmental stages of the fluke have to adapt during the course of its life cycle. Gene ontologies of the differentially expressed genes revealed a wide range of functions and processes. In addition, stage-specific, differentially expressed genes were identified that were involved in numerous biological pathways and functions including calcium signalling, sphingolipid metabolism and parasite defence.

**Conclusion:**

The findings provide a comprehensive database of gene expression in an important human pathogen, including transcriptional changes in genes involved in evasion of the host immune response, nutrient acquisition, energy production, calcium signalling, sphingolipid metabolism, egg production and tegumental function during development. This resource should help facilitate the identification and prioritization of new anti-schistosome drug and vaccine targets for the control of schistosomiasis.

## Background

**Figure 1:**
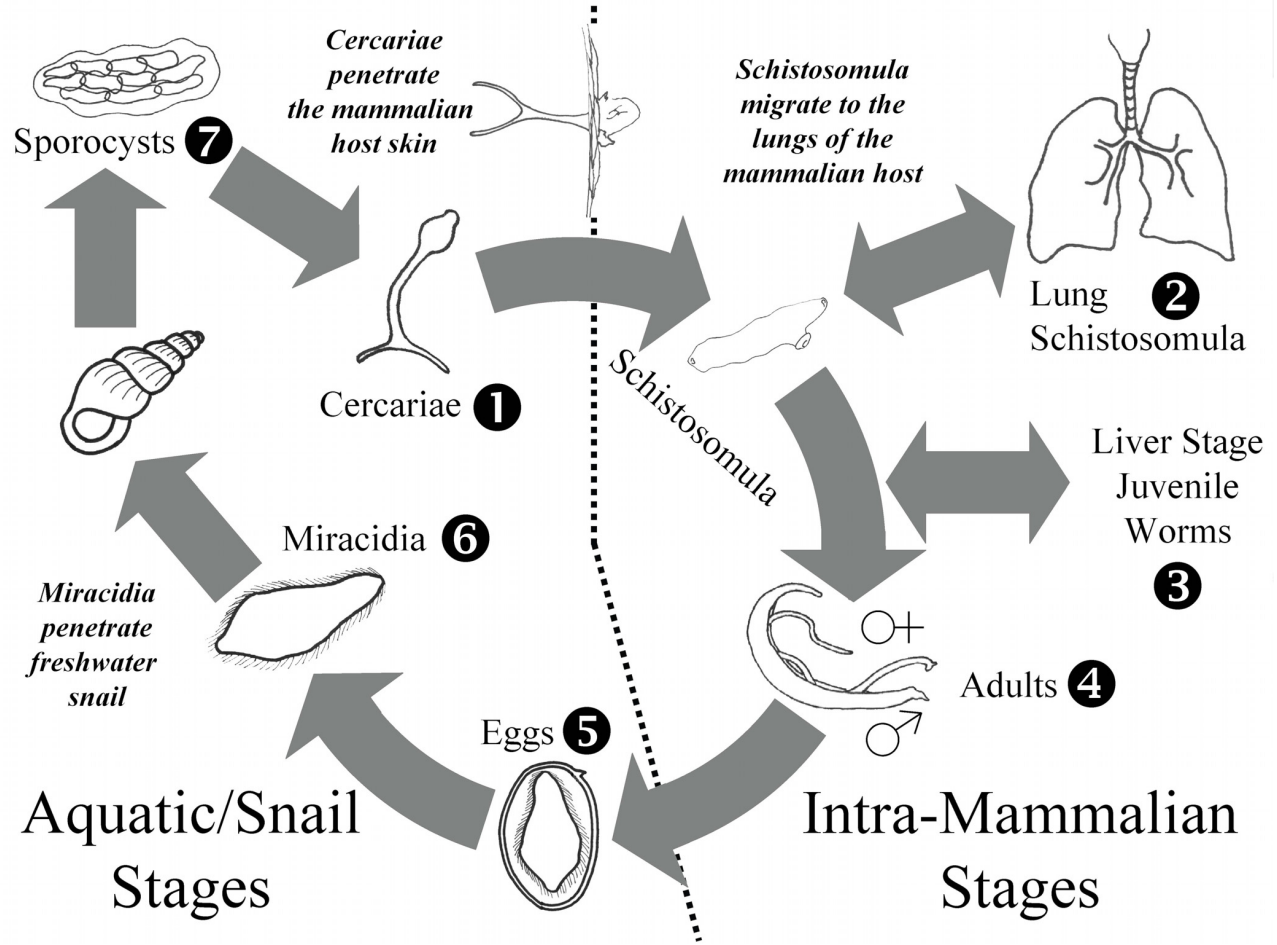
**The complex lifecycle of *Schistosoma japonicum *involves distinct free-living and parasitic stages (see text for details)**. The numbers indicate the seven developmental stages investigated by microarray and real time PCR analysis.

Schistosomiasis afflicts ~200 million people in 76 countries [1]. The disease is caused by infection with blood flukes of the genus *Schistosoma *and depending on the invading species, is characterised clinically by chronic hepatic and intestinal fibrosis, portal hypertension, anaemia and calcification of the urinary tract. The parasitic worms have a complex developmental cycle that involves infection of freshwater intermediate molluscan hosts and the blood stream of mammals (Figure 1). Schistosome infection results from direct contact with fresh water contaminated by free-swimming larval forms of the parasite known as cercariae. Cercariae penetrate human skin, shed their tails, releasing schistosomula which enter capillaries and lymphatic vessels en route to the lungs. After several days, the male and female juvenile worms migrate to the portal venous system, where they mature and unite. Adult worm pairs then migrate to the veins of the intestines, in the case of *Schistosoma mansoni *and *S. japonicum*, or the bladder with *S. haematobium*. Egg production commences four to six weeks after infection and continues for the life of the worm – usually three to five years. Eggs pass from the lumen of blood vessels into adjacent tissues, and many then pass through the intestinal or bladder mucosa and are shed with the faeces or urine. The life cycle is completed when the eggs hatch, releasing miracidia that, in turn, infect specific freshwater snails. After two asexual generations within the snail, mother followed by daughter sporocysts, cercariae are released.

Schistosomes undergo dramatic changes in gross morphology and cellular composition and specialization during their transition from free-swimming cercariae in freshwater to mature adult worms, which reside in the ostensibly inimical environment of the mammalian host blood stream [2]. In previous microarray studies, several hundred cDNAs from *S. japonicum *and *S. mansoni *[3,4] or a few thousand [5] oligonucleotides based on *S. mansoni *sequences, were used to identify sex-, developmental stage- and strain-specific genes, providing a clear indication of the value and power of microarray analysis for studying the biology of schistosomes. A subsequent study [6] examined 3,088 contigs or singletons across seven life cycle stages of *S. mansoni *early liver worms, adult worms, eggs, daughter sporocysts, cercariae, and day 2 and day 7 schistosomula produced by mechanical transformation of cercariae followed by *in vitro *culture. A new 22,575 feature 60-mer microarray was deployed to investigate gene expression patterns between and within discrete Chinese and Philippine strains of *S. japonicum *[7] and to demonstrate stage-associated gene expression between mature adult worms and lung schistosomula from amplified *S. japonicum *mRNAs [8]. Recently, [9] investigated gene expression profiles in cercariae, sporocysts, mechanically transformed schistosomula and paired male and female adult worms of *S. mansoni*. Other methods have also been successfully employed to investigate gene expression changes in schistosomes, including the use of serial analysis of gene expression (SAGE) to study *S. mansoni *[10]. In the present study, we deployed a custom designed oligonucleotide microarray to profile gene expression throughout the development of *S. japonicum*, the Asian blood fluke, with hybridizations of RNAs from seven developmental stages of the parasite – lung stage schistosomula, 4-wk-old immature female and male worms, sexually mature male and female worms, eggs, miracidia, sporocysts and cercariae. The findings of this study establish baseline transcriptional information of the developmental biology of this important human pathogen, and identified stage-specific, differentially expressed genes involved in numerous biological pathways and functions including calcium signalling, sphingolipid metabolism and parasite defence.

## Results

### Filtering of microarray data to identify stage enriched genes

All microarray hybridisations were performed in triplicate with normalisations and filtering of data performed using GeneSpring GX. Normalised data were filtered for each replicated data point for each of the 38,444 probes (19,222 contigs) on the schistosome microarray (Additional file 1). Data points were filtered to preserve signals that were flagged during the feature extraction process as *Present or Marginal *in all hybridisations; this resulted in the retention of 7,132 probes (4,371 contigs). Next, ANOVA applied to the data identified 6,465 probes (4,104 contigs) differentially expressed due to transcriptional changes throughout the *S. japonicum *lifecycle.

A final cut-off was applied to the microarray data generating lists of genes that were ≥ 2 fold (relative to the median intensity) in each lifecycle stage; these combined lists consist of 4,443 probes/3263 genes represent stage-enriched gene transcripts. These lists comprised of a subset of 1,782 unique genes or contigs, and were subjected to further annotation or *in silico *characterisation. The numbers of 2 fold or 5 fold or higher enriched genes for each individual developmental stage or lifecycle grouping is summarised in Table 1 (individual lists are available as Additional file 2). Examples of differentially expressed genes for each lifecycle stage are presented in Table 2.

**Table 1:** Number of upregulated genes in *S. japonicum *determined by ANOVA.

	**Normalised Intensity/Median Intensity of All Stages**			
	**2 fold**		**5 fold**	
**Lifecycle Stage**	**Probes**	**Genes**	**Probes**	**Genes**
Eggs	1312	931	136	103
Miracidia	399	306	195	162
Sporocysts	327	244	95	78
Cercariae	899	629	106	72
All Aquatic/Snail Stages	80	62	11	9

Lung Schistosomula	366	296	133	117
Juvenile Males	102	81	35	27
Juvenile Females	197	149	45	39

Adult Males Combined weeks 6–7	307	217	70	54
All Males Parasites + juvenile	79	62	21	16
Adult Females combined weeks 6–7	241	184	54	47
All Females Parasites + juvenile	138	106	47	42

**Total**	**4447**	**3267 (1782)**	**948**	**766**

The intensity for each development stage was normalised to the median intensity of all the developmental stages examined for each gene, thus providing a relative fold change. The number of probes or genes enriched 2- or 5-fold is presented. The 1782 value in parenthesis in the Total row, refers to the number of non-redundant genes enriched 2-fold or higher.

**Table 2:** Some of the highly enriched transcripts from the different developmental stages of *S. japonicum*.

**Lifecycle Stage**	**Probe**	**Nucleotide Description/Protein Homology**	**Fold Δ**
***Egg***	Contig7259	/SJCHGC02876/aquaporin 9	100.91
	Contig8558	S. mansoni calcium-binding protein/calmodulin	99.13
	Contig7271	S. mansoni anti-inflammatory protein 16/SJCHGC05573	49.15
	Contig3014	solute carrier family 37 (glycerol-3-phosphate transporter)	2.35
	Contig7936	S. mansoni eukaryotic translation initiation factor 2	2.30
	Contig4628	A. thaliana dynamin-like protein 6 (ADL6).	2.07
	
			
***Miracidium***	Contig1657	,/insulin-like peptide receptor ilp-r	79.25
	Contig714	S. mansoni for Sm10 protein/dynein light chain 2	19.99
	Contig7933	S. mansoni calponin homolog,/SJCHGC02977	18.55
	Contig5142	solute carrier family 7 (cationic amino acid)	16.25
	Contig3311	monocarboxylate transporter	12.58
	Contig8754	cytochrome c oxidase subunit i	2.76
	Contig4272	S. japonicum for serine-enzyme/---NA---	2.03
	
			
***Sporocyst***	Contig714	S. mansoni for Sm10 protein/dynein light chain 2	79.73
	Contig6889	S. japonicum clone ZZZ32/plexin domain containing 2	27.99
	Contig5142	SJCHGC04289/solute carrier family 7 member 8	8.48
	TC16140	pyruvate kinase	3.55
	Contig8948	ribosomal protein s14	3.02
	Contig5652	udp-glucose dehydrogenase	2.95
	Contig1610	ribosomal protein l31	2.54
	Contig6944	leucine zipper-ef-hand containing transmembrane protein 1	2.02
	
			
***Cercaria***	Contig8837	S. japonicum clone ZZD363/glutamine synthetase	11.12
	Contig5222	S. japonicum thioredoxin,/mgc80314 protein	8.73
	Contig6136	Canis familiaris endothelin B receptor/SJCHGC09785	7.27
	Contig5932	o-linked n-acetylglucosamine transferase	4.13
	Contig1574	/NADH dehydrogenase subunit 5 [Strongyloides stercoralis]	3.69
	Contig7759	S. japonicum cytochrome c oxidase 3	3.11
	Contig7070	actin binding1b	2.71
	Contig4282	dynamin 1	2.49
	Contig4204	/cytochrome c oxidase subunit III [Siphonodentalium lobatum]	2.43
	Contig690	/Cytochrome C oxidase, mono-heme subunit [Shewanella denitrificans ]	2.34
	Contig8895	S. japonicum actin mRNA, complete cds/actin	2.32
	Contig6947	amp-activated protein kinase	2.26
	Contig7330	S. japonicum cytochrome C oxidase copper chaperone	2.23
	Contig3578	protein phosphatase 1b magnesium-beta isoform	2.12
	Contig1640	esterase a	2.06
	Contig1629	histone deacetylase 3	2.06
	TC17311	glycogen synthase kinase 3 alpha	2.00
	
			
***Lung Schistosomula***	Contig4998	/ef-hand calcium binding protein	9.68
	Contig7933	S. mansoni calponin homolog,/SJCHGC02977	5.37
	Contig7060	S. japonicum polyubiquitin (UBC)	4.23
	Contig6917	secretory carrier membrane protein 2	3.90
	TC17030	centrin 3	3.87
	Contig6538	S. japonicum for paramyosin,/paramyosin	3.27
	Contig7916	calreticulin	2.46
	Contig6517	S. mansoni heat shock protein 70	2.40
	TC14572	synaptojanin 1	2.05
	
			
***Juvenile Male***	TC13825	SJCHGC01839/glioma pathogenesis-related protein	8.82
	Contig4272	S. japonicum for serine-enzyme/---NA---	5.00
	Contig8395	S. mansoni 200 kDa surface protein,/SJCHGC09596/	3.30
	Contig8253	thymosin beta	2.79
	Contig8718	glycosyltransferase 1 domain containing 1	2.37
	Contig8946	S. japonicum fatty acid binding protein	2.21
	Contig8017	annexin a7	2.02
	
			
***Juvenile Female***	TC9476	putative ferredoxin reductase; Mesorhizobium loti/	58.52
	Contig7517	SJCHGC00284/epididymal secretory protein e1	10.47
	Contig8731	S. japonicum clone ZZD10/SJCHGC06317/cd63 antigen	6.36
	Contig5142	solute carrier family 7 (cationic amino acidy+ system)member 8	5.27
	Contig2104	valosin containing protein	2.57
	Contig7465	adenylosuccinate synthase like 1	2.49
	TC11333	DNA mismatch repair protein MSH2 – African clawed frog	2.29
	Contig5394	novel transmembrane amino acid transporter protein	2.26
	Contig3981	thioredoxin peroxidase	2.09
	
			
***Adult Male***	Contig7688	S. japonicum dynein light chain 3 (DLC3)	13.31
	Contig7484	S. japonicum clone ZZZ342/cd38 antigen	10.26
	Contig4443	solute carrier family 1 (glial high affinity glutamate transporter)	6.01
	Contig8927	S. japonicum clone ZZZ66/SJCHGC00511/cathepsin f	4.85
	Contig7921	S. japonicum cathepsin B endopeptidase/cathepsin b	4.57
	Contig4208	H. sapiens clone RP11-124P23/SJCHGC05752/paramyosin	4.27
	Contig4684	/SJCHGC01577/annexin 5	3.75
	Contig7122	S. mansoni tegumental antigen (Sm8)/SJCHGC00665	3.54
	Contig8822	S. japonicum actin	3.30
	Contig8859	myosin heavy chain	3.24
	TC7585	fimbrin {S. mansoni}, complete	3.19
	TC17273	alkaline phosphatase	3.18
	Contig2404	sperm associated antigen 6	2.49
	Contig8527	solute carrier family 2 (facilitated glucose transporter)member 5	2.37
	Contig6302	clathrin coat assembly protein ap19	2.31
	Contig1963	S. mansoni glutathione S-transferase	2.20
	
			
***Adult Female***	Contig5142	SJCHGC04289/solute carrier family 7 member 8	11.85
	Contig8731	S. japonicum clone ZZD10/SJCHGC06317/cd63 antigen	9.39
	Contig7312	/SJCHGC05381/transmembrane 9 superfamily member 2	5.40
	Contig7830	/SJCHGC02408/ferric-chelate reductase 1	3.94
	TC8025	ribosomal protein s27-like	3.74
	TC11576	ribosomal protein l37	3.59
	TC7041	ribosomal protein l7a	3.32
	Contig7888	folate-binding protein 1	3.31
	TC14195	60s ribosomal protein l39	3.25
	Contig8147	S. mansoni receptor for activated PKC	3.06
	Contig2978	microtubule associated serine threonine kinase-like	2.69
	TC11333	DNA mismatch repair protein MSH2 – African clawed frog	2.60
	Contig7235	calmodulin	2.52
	TC7616	60s acidic ribosomal protein p2	2.46
	Contig8062	phosphogluconate dehydrogenase	2.36
	Contig1324	copper chaperone	2.35
	TC12062	glucosidase 1	2.15
	Contig3791	asparagine-linked glycosylation 6 homolog	2.10
	Contig8420	arginase ii	2.03

### Hierarchal clustering to provide overview of developmental changes

**Figure 2:**
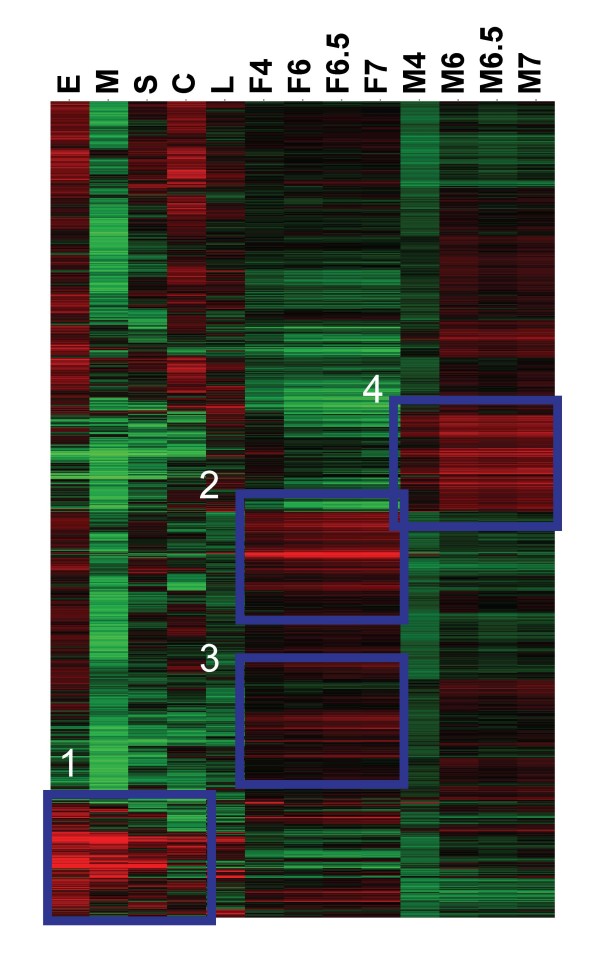
**Hierarchal clustering by gene and developmental stage**. Four clusters of up-regulated genes were identified: 1, Aquatic/snail stages; 2 and 3, Adult female worms; 4. Adult male worms. Gene expression is shown in the heat map as up-regulated (Red) down-regulated (Green) or no change (Black). E, eggs; M, miracidia; S, sporocysts; C, cercariae; L, lung schistosomula; F4, juvenile females; M4, juvenile males; F6, F6.5, F7, adult female worms analysed at 6, 6.5 and 7 weeks post-cercarial challenge; M6, M6.5, M7, adult male worms analysed at 6, 6.5 and 7 weeks post-cercarial challenge.

Hierarchal clustering was employed to investigate gene expression profiles in each developmental stage. Figure 2 is a heat-map that presents the hierarchal clustering performed on genes, identified by ANOVA, with clustering for both gene and developmental stages; in Figure 2, up-regulation is shown in red, down-regulation in green, whereas the black lines indicate absence of up- or down-regulation. As could be predicted, based on the marked changes in morphology and the discrete environmental changes encountered by the different developmental stages, there were clear differences in transcription (up or down regulation) among the ~6,500 probes examined. For example, considerable enrichment of gene expression was evident in the egg compared with the miracidial stage (Figure 2; Table 1). Among the developmental stages, four prominent clusters of up-regulated genes were evident these were in (1) aquatic and snail stages, (2) adult female worms [with corresponding down-regulation in males], (3) adult female worms [with no down-regulation in males], and (4) adult males.

### Blast2Go and gene ontology analysis to provide overview of functions and process

**Figure 3:**
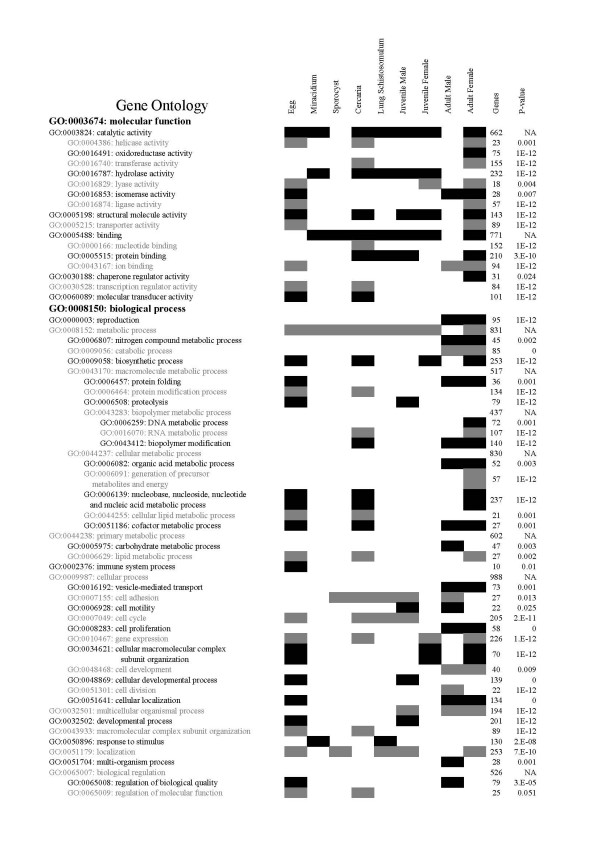
**Gene Ontology (GO) analysis showing the correlation between GO categories and microarray expression data calculated using ErmineJ software**. The GO annotations with parent-child analysis are presented on the left. Contributions to each of the categories from each lifecycle stage (2-fold or higher gene expression) are shaded. The overall number of genes in each category represented on the microarray and the correlation p-value associated with the entire lifecycle are shown on the right. NA, the parent category is significant but a child category is not. Genes expressed in adult parasites from weeks 6–7.

The 1,782 genes that were identified as being enriched 2 fold in any given lifecycle stage were subjected to protein translational blast and gene ontology (GO) batch analysis. This presented a further overview of the gene ontologies that are modulated during the *S. japonicum *lifecycle; two of the three major categories, Biological Process and Molecular Function, are presented in Additional file 3. This information was used to supplement the already known GOs based on nucleotide sequence previously published [11]. To gain a more complete overview of the GO categories that are modulated during the *S. japonicum *lifecycle we used the software ErmineJ to correlate this extended list of GOs with microarray fold change data. Presented in Figure 3 are the prominent GOs associated with the lifecycle. In the Biological Processes category were developmentally expressed genes involved in cellular or primary metabolism, cellular communication and biosynthesis, while outstanding among the Molecular Function category were developmentally regulated genes involved in the binding of proteins, nucleotides, nucleic acids and enzymes such as hydrolases and transferases. During development of *S. japonicum *genes in the GO category of Biological Processes were most frequently involved in metabolism whereas those categorised within Molecular Functions were most frequently involved in binding and enzyme activity. These trends were, in general, similar to the GO findings observed previously in EST transcriptome studies of both *S. japonicum *[12] and *S. mansoni *[13].

Examples of KEGG pathways identified by BLAST2GO analysis, ie pathways that contain multiple genes involved in metabolism/anabolism are presented in Supplementary Figures 2-4. Genes within the Pentose Phosphate, Calcium Signalling and Sphingolipid Metabolism pathways were expressed at higher levels in several of the *S. japonicum *lifecycle stages. These included genes encoding fructose-bisphosphate aldolase (Contig8868), sarcoplasmic reticulum ATPase calcium pump (Contigs 3653, 4586), and sphingomyelin phosphodiesterase (Contig7899).

### Microarray findings are supported by real time PCR

**Figure 4:**
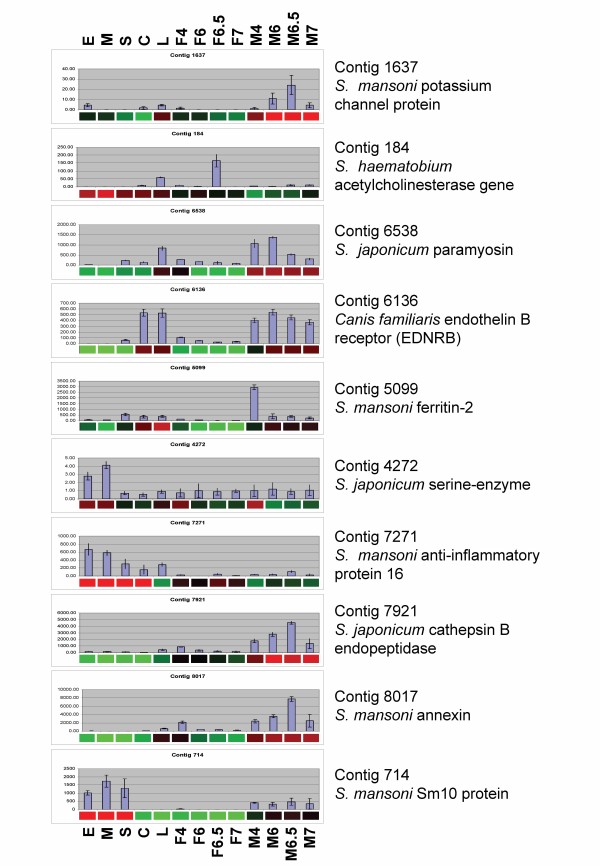
**Validation of selected transcripts in different developmental stages of *S. japonicum***. The real time PCR analysis is presented as bar graphs and is shown in copy number for each gene and stage. The corresponding microarray gene expression data are presented below the bar graphs as heat maps, with up-regulated genes shown in red, down-regulated genes shown in green and unchanged genes shown in black. E, eggs; M, miracidia; S, sporocysts; C,: cercariae; L, lung schistosomula; F4, juvenile females; M4, juvenile males; F6, F6.5, F7, adult female worms analysed at 6, 6.5 and 7 weeks post-cercarial challenge; M6, M6.5, M7, adult male worms analysed at 6, 6.5 and 7 weeks post-cercarial challenge.

NADH-ubiquinone reductase was selected as a housekeeping gene for real time PCR analyses as this gene was constant in all 39 microarray hybridisations performed. A subset of 10 differentially expressed genes, representative for each of the life cycle stages examined, were subjected to verification by real time PCR (Figure 4). The majority of genes that were shown differentially expressed by the microarray analysis correlated well with the results obtained by real time PCR. Figure 4 outlines the 10 genes examined by real time PCR (bar graphs) and presents also the corresponding microarray signal detected (underlying heat map bar).

### Genes within clusters identified by hierarchal clustering

Examples of genes upregulated in the aquatic/snail lifecycle stages included a gene encoding an anti-inflammatory protein Sj16 (Contig7172) which was enriched (11-fold upregulated) in cercariae, eggs (49-fold upregulated), miracidia (38-fold upregulated) and sporocysts (13-fold upregulated); it was also enriched in lung stage schistosomula (13-fold upregulated). A homologue (Contig8558) of the *Schistosoma mansoni *calcium-binding calmodulin was also upregulated in several stages, including eggs (99 fold), miracidia (73 fold) and sporocysts (4 fold). Genes encoding putative dynein proteins were differentially expressed in the aquatic/snail stages including Sm10 (Contig714) which was upregulated in eggs (32-fold), miracidia (20-fold) and sporocysts (80-fold); Sj21.7 (Contig2186) mirrored this expression pattern with gene expression enriched in eggs (24-fold), miracidia (17-fold) and sporocysts (5.6-fold). Genes involved in mitochondrial-linked energy metabolism were upregulated predominantly in eggs and the motile cercariae with other aquatic/snail stages also enriched for these genes. Examples of enriched mitochondrial genes included Contig8754 and Contig7759 encoding cytochrome c oxidase subunit I and cytochrome c oxidase 3, respectively. Enrichment of these two genes occurred in eggs (3 & 5.6 fold), miracidia (2.2 & 2.3 fold), sporocysts (2.7 & 1.2 fold) and cercariae (3.4 & 3.1 fold).

Adult female *S. japonicum *expressed high levels of a suite of genes reflecting well recognised biological functions associated with this stage. These included genes involved in iron metabolism including ferric-chelate reductase 1 (Contig7830, 3.9 fold) and a putative ferredoxin reductase (TC9476, 134 fold). Transporters were also enriched including Contig5142 (solute carrier family 7, 11.8 fold), Contig7944 (sodium-dependent amino acid transporter, 3.8 fold), TC8509 (solute carrier family 25, 2.7 fold), Contig5284 (solute carrier family member b1, 2.6 fold) and Contig3928 (novel protein vertebrate udp-galactose transporters, 2.5 fold). Multiple ribosomal genes were over expressed (Table 2) reflecting the high protein requirements of the female worm associated with egg production.

Notable genes upregulated in adult male *S. japonicum *were those involved in molecular transport including Contig4443 (solute carrier family 1, 6 fold) and Contig8527 (glucose transporter protein GTP1, solute carrier family 2, 2.4 fold). Other genes enriched in adult males included the endopeptidases cathepsin B (Contig7921, 4.6 fold) and cathepsin F (Contig8927, 4.8 fold); components of vesicle mediated transport for both endocytosis-clathrin coat assembly protein ap19 (Contig6302, 2.4 fold), adaptin-related protein 2 (TC7859, 2.6 fold) and exocytosis-syntaxin 16 (Contig7586, 1.5 fold). Genes involved in carbohydrate metabolism and glycolysis, including glycosyltransferase 1 (Contig8718, 4.1 fold) and pyruvate dehydrogenase (Contig4093, 2.7 fold), were also enriched. Further examples of enriched genes for each of the lifecycle stages examined are presented in Table 2.

### Comparison of lifecycle stages

**Figure 5:**
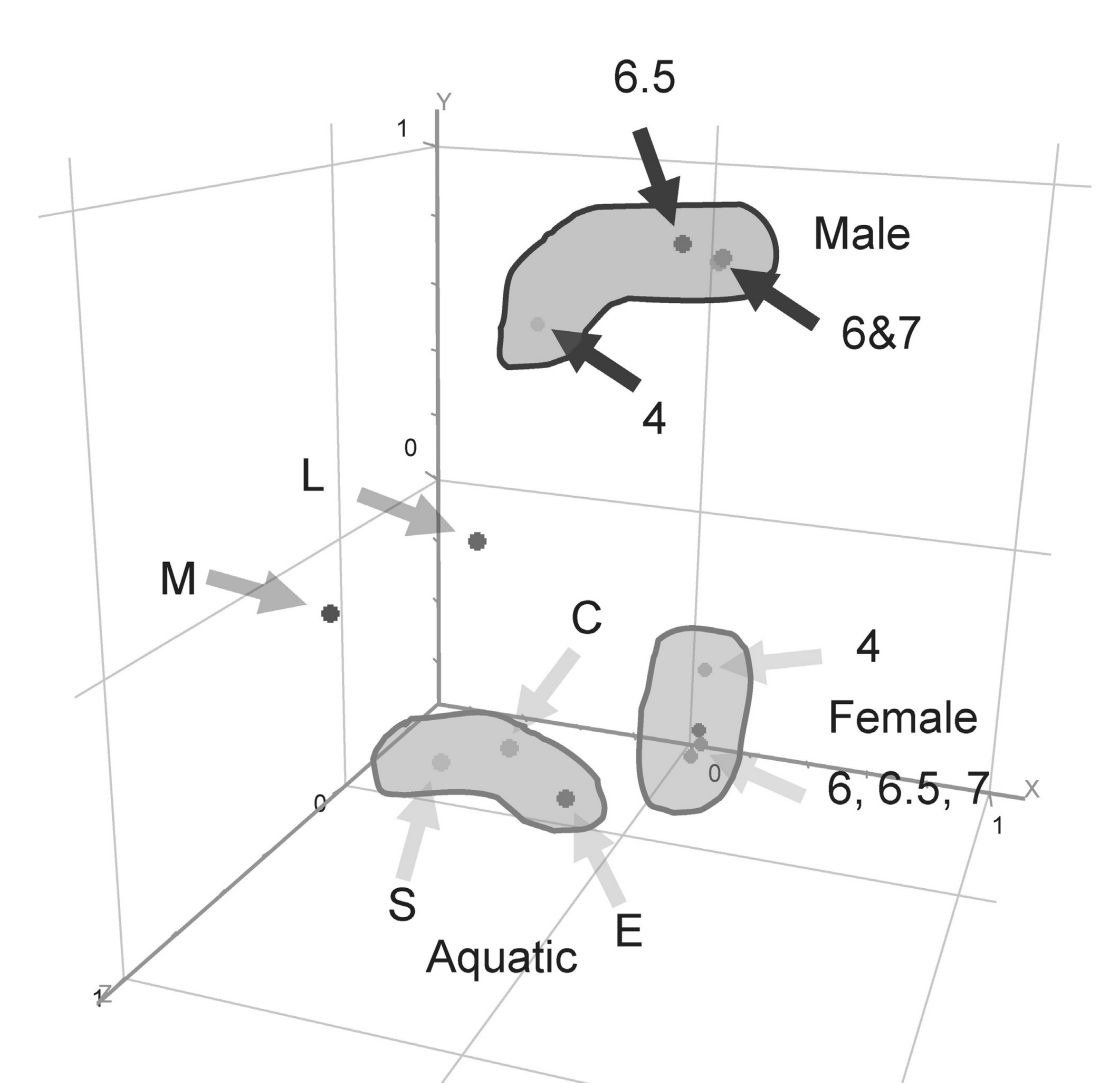
**Principal component analysis of the ANOVA showing an overview of differentially expressed genes in the different developmental stages of *S. japonicum *analysed**. E, eggs; M, miracidia; S, sporocysts; C,: cercariae; L, lung schistosomula; F4, juvenile females; M4, juvenile males; F6, F6.5, F7, adult female worms analysed at 6, 6.5 and 7 weeks post-cercarial challenge; M6, M6.5, M7, adult male worms analysed at 6, 6.5 and 7 weeks post-cercarial challenge.

Principal component analysis (PCA) was employed to establish relationships of the gene expression profiles among the different developmental stages of *S. japonicum*. The PCA demonstrated clearly that stages that were temporally related (e.g. sporocyst to cercaria) exhibited similarities in gene expression (Figure 5). In general, the developmental stage expression profile, based on PCA, clustered into three major groupings: (1) adult males, (2) adult females, and (3) aquatic and snail stages comprising eggs, sporocysts and cercariae. Furthermore, a clear change of transcript profile was evident within the male and female adult worms, as the females mature from pre-egg laying to egg-laying status at four to six weeks post-infection of the mammalian host. By contrast, the schistosomulum (mammalian lung) and miracidium (ciliated, free-living aquatic larva) did not display obvious associations or grouping with any of the other stages.

Gene expression profiles for 7 week-old adult males of *S. japonicum *examined by PCA and ANOVA analysis revealed limited variation between biological replicates, ie worms obtained and examined from independent infections at different times (not shown). This was also the case when pools of biological replicates of adult male worms were analysed (n = 4) (not shown). After filtering the data initially for flags, 10,765 probes were further filtered for ANOVA. At the default settings, no genes were identified as being significantly variable (p-value = 0.05) between biological replicates; only 27 genes were identified as variable after increasing the error cut-off rate to 18% (the rate at which the identified genes would be expected to pass the restriction by chance). This indicated that a very low level of variation in gene expression occurred between biological replicates resulting from parasite samples obtained from separate infections.

## Discussion

Microarrays provide a powerful platform to monitor developmental changes in the transcriptome of an organism and a foundation for studies of gene regulation and proteomics analysis [14]. This study provides and compares the baseline transcriptional levels of the majority of genes in discrete developmental stages of *Schistosoma japonicum *including those within the definitive (eggs, lung schistosomula, juvenile and adult male and female worms) and intermediate (sporocysts) hosts as well as the aquatic, free-swimming miracidia and cercariae. Gene transcription studies of many metazoan parasites, including the schistosomes, have been disadvantaged because these pathogens cannot be maintained in culture and/or are only available in limited quantities for analysis. The deployment of gene microarrays can help obviate these challenges since only small quantities of scarce microbial tissues are required for preparation of targets for microarray analysis.

Our findings present the first global overview of differential gene expression among the major developmental stages of this schistosome. The final fold cut-off was applied to the microarray data, thus generating lists of genes that were ≥ 2 fold (relative to the median intensity) in each lifecycle stage; these combined lists consist of 4,443 probes/3263 genes representing stage-enriched gene transcripts. We choose this arbitrary level due to its general acceptance in many other similar microarray studies. However it is known that statistical variability is primarily chip specific [14,15], and the selection of a fold change of ≥ 2 can be related back to previous experiments with the same chip [7], thus providing confidence in our findings.

As illustrated in Figures 2 and 3, similarities and relationships were apparent in the gene expression of several of the developmental stages. In particular, gene expression profiles were more similar within the 4, 6, 6.5 and 7 week old adult parasites, although there was an apparent change in expression from week four (pre-egg laying in female worms) to week six onwards (after the commencement of egg release). This similarity in gene expression likely reflects parasitism of the mammalian blood vessels by the schistosome and the discrete differentiation and development of the males and females [4,7,16,17]. The eggs, sporocysts and the cercariae also shared similarities in gene expression profiles. These similarities likely reflect adaptations to the aquatic environment and parasitism of the snail host, but are somewhat surprising in regards to the eggs, given that these were recovered from mouse livers.

It is not straightforward to compare the present findings with the earlier reports of microarray-based gene expression in schistosomes given that many of the previous investigations focused on *S. mansoni*. Nonetheless, we have been able to make some general comparisons, including demonstration of the sex-specific nature of some important developmental changes in *S. japonicum*. For example, upregulated gene transcription for S-transferase SM28 antigen (TC10486) [Contig6578 (glutathione s-transferase, 1.7–2 fold), dynein 8 kDa light chain flagellar outer arm (TC8189) (8 kDa outer arm dynein light chain 1.8–2.3 fold) and TC10493 Cathepsin B1 isotype 1 (3.2–4.0 fold) was evident in adult male worms. Similar enrichment of these genes was shown in sporocysts and adults of *S. mansoni *[9] but, as we separately investigated the sexes of adult *S. japonicum*, we were able to show that this up-regulation was male-specific.

However, a direct correlation of small subset of probes originally designed from *S. mansoni *EST and present on both microarrays (TC probes) was possible. Additional file 4 shows that of the 20 TC probes which had data from *S. japonicum *and *S. mansoni*, only 5 probes could be correlated as up-regulated to the same lifecycle stages for both species (shaded in the Table). This comparison highlights the difficulty of inter-species comparisons using microarray technology, a limitation that is compounded by the lack of complete genomes for both *S. japonicum *and *S. mansoni *and the absence of comprehensive bioinformatics comparisons at the genomic level. However, the current study of *S. japonicum *has provided a more comprehensive overview of the developmental biology of schistosomes in terms of the coverage of the transcriptome through the size of the microarray used, and also the number of lifecycle stages examined including separate adult sexes, *in vivo *derived lung schistosomula, miracidia and the juvenile pre-egg producing parasite. Additionally the study has also used analysis methods to cluster multiple genes to KEGG pathways not applied previously in schistosome microarray studies. This analysis has further highlighted stage specific functions and provides insights into transcriptional changes of schistosome genes involved in evasion of the host immune response, nutrient acquisition, energy production, calcium signalling, sphingolipid metabolism, egg production and tegumental function during development (as is discussed further below).

A brief description of some of the other genes developmentally expressed in the various lifecycle stages of *S. japonicum *follows. Sm16 is an anti-inflammatory glycoprotein first identified in adult *S. mansoni *[18] that was shown by immunoelectron microscopy to be localised in cercariae in the acetabular glands and in subtegumental cell bodies packed with membranous vesicles [19]. Sm16 acts as a lipid binding protein that is taken up by mammalian cells via endocytosis and acts within the cytoplasm to induce apoptosis [20]. IL-1a is stimulated by Sm16 which via keratinocytes leads to a decrease in the number of lymphoproliferative cells that are recruited, presumably during the penetration of the mammalian host, by the invading skin schistosomula [21]. Sm16 may aid *S. mansoni *in penetration of the mammalian host [20] and our results support this hypothesis as the transcript of the *S. japonicum *homologue (Sj16; Contig7172) was 11-fold enriched in cercariae. However, the microarray and/or real-time PCR analysis indicated that even higher levels of the gene were expressed in the egg (49-fold upregulated), miracidium (38-fold upregulated), sporocyst (13-fold upregulated), and lung stage schistosomulum (13-fold upregulated). This expression profile suggests an additional intra-mammalian role for the product of this gene perhaps as a defence mechanism against the host immune system in both the invading cercariae, the lung schistosomulum and the egg, which during the chronic stage of infection induces granuloma formation and fibrogenesis [22]. The high levels of Sj16 expression in the miracidium and sporocyst may reflect a role in the penetration of the molluscan host and in protection of the sporocyst from snail innate immunity.

The microarray and real time PCR analysis revealed elevated levels of expression of paramyosin (Contig6538) in the adult male (microarray 2.5–3-fold upregulated) and the lung schistosomulum (real time PCR 3.7-fold upregulated). These profiles correlate with the well documented functions for paramyosin in schistosomes as a structural component of smooth muscle fibres and as an immunomodulator of the host immune response through its binding to the immunoglobulin Fc region and its inhibition of complement activation [23-25]. The highly developed musculature of the adult male [26] supports the female adult worm *in copula*. The localisation of paramyosin on the surface of the lung schistosomulum [23] was the first indication of the developmental significance of the expression of this protein. Immunological and biochemical findings [24,27] have further emphasised the importance of paramyosin in protecting schistosomes from immune attack by binding proteins of the complement pathway [28].

The antioxidant, thioredoxin is present in cercariae, schistosomula, eggs and both male and female adults of *S. mansoni *[29]. Thioredoxin is secreted by eggs of *S. mansoni *[30], and likely is implicated in granuloma formation within the liver of the mammalian host [31]. Our gene microarrays revealed that *S. japonicum *thioredoxin (Contig3028) expression was up-regulated in the egg (2.4-fold), thereby confirming its importance in this stage and also the adult male (2.6–2.7-fold). The antioxidant properties of thioredoxin would be expected to protect the schistosome from reactive oxygen species released by host inflammatory responses

Calponin is a calcium binding protein that inhibits the ATPase activity of smooth muscle myosin necessary for long-term contractions. It is expressed predominantly within the muscle fibres of adult male and female *S. japonicum *[32]. Further localization studies have indicated that expression also occurs within the stratified muscle of the tail and smooth muscle of the head region of cercariae [33]. The current microarray results support these observations but also showed enrichment of calponin (Contig7933) in lung schistosomula (5.4-fold) and the aquatic/snail stages (egg, 12.8-fold; miracidium, 18.5-fold; sporocyst, 6.2-fold; cercariae, 3.4-fold). This likely reflects general upregulation of muscle-related genes as schistosomes have significantly increased muscle deposition during development [34].

Annexins bind phospholipids in a calcium-dependent manner and are thought to reduce inflammation via the suppression of phospholipase A2 in a similar manner to glucocorticoids [35]. Other predominant functions of annexins include the organisation of cellular structures, signal transduction, cellular growth and modulation of intracellular calcium while acting as atypical calcium channels [35]. Annexins are a well known component of the adult schistosome tegument as shown by surface biotinylation and proteomics studies [36]. Our microarray and real time PCR analysis revealed that annexin (Contig8017) expression was raised in lung schistosomula (1.3–1.4-fold) and adult males (2-8-3.3-fold) of *S. japonicum*. Both of these lifecycle stages would benefit from the anti-inflammatory properties of annexin due to the immune response generated in the lungs, and the relatively large number of tegumental proteins present on the adult male parasite that may incite an immune response [37].

In contrast to what is currently known regarding calcium channels in schistosomes, including sub-unit structure, cellular distribution and interactions with the anti-schistosomal drug praziquantel [38], there is limited knowledge of the biology of potassium channels in these worms. What is known is that they are localised to the nervous system and musculature of adult *S. mansoni *[39]. This is supported by the findings presented here as both the microarray and real time PCR analysis showed highest expression (12 fold enriched) of the potassium channel gene (Contig1637) in adult male *S. japonicum*, which has an extensively developed musculature [26].

Sm21.7 has sequence similarity to dynein light chains (DLCs), it is one of a family of EF-hand containing parasite proteins and is also a major non-parasite allergen [40]. Little is known of the function of Sm21.7 but its transcript was originally shown to be present in the sporocyst, mechanically transformed schistosomulum and adult *S. mansoni *[41]. Subsequently, it has been localised to the adult worm tegument, it is expressed in eggs, adults and cercariae and is known to stimulate the mammalian immune system since it is constantly released by eggs trapped in liver granulomas [40]. Here, the results of the developmental expression studies on the *S. japonicum *homologue, Sj21.7, mirrored in part, the previous studies on Sm21.7 but transcripts for Sj21.7 (Contig2186) were enriched predominantly in the egg (24-fold), miracidium (17-fold) and sporocyst (5.6-fold) with limited expression in the intra-mammalian stages, possibly indicating differences in functional expression between the two schistosome species. Another gene exhibiting a similar expression profile to Sj21.7, as determined by the microarray analysis and real time PCR was Sm10 (Contig714), being upregulated in eggs (32-fold), miracidia (20-fold) and sporocysts (80-fold). This gene encodes another DLC that has been localised to the tegument [37] and is strongly immunogenic [42]. The microarray analysis suggests that the DLCs of *S. japonicum *may have a greater requirement for microtubule motility during the aquatic developmental stages compared with the intra-mammalian stages. This observation is particularly pertinent since the highest expression levels of Sm10 were found in miracidia which utilise cilia for locomotion. While Sm10 has not yet been immunolocalised in the miracidium stage, there is evidence for a role of dyneins in the cilia and flagella of other mammalian and non-mammalian (*C. elegans*) systems [43]. Sm10 has homology to members of the LC8 family of DLCs, of which one member, DYNLL1, is up-regulated in mammalian testes and lung, two tissues which have considerable numbers of cilia or flagella present [44].

The *S. japonicum *transcript encoding the homologue of sperm associated antigen 6 (Spag6) (Contig2404) is likely a component of tubulin structures and was enriched in both the egg (2.4-fold), the adult stages (1.6–2.6 fold) and to a lesser extent, juvenile males (1.5-fold). Spag6 is the murine orthologue of *Chlamydomonas *PF16, a gene that is a component of the flagella central apparatus [45], so the enrichment of this gene in adult male *S. japonicum *likely reflects a role in reproduction, probably in sperm function. Its involvement in eggs is less clear but may reflect a structural role associated with microtubules and development of the miracidium.

Sphingolipids represent a class of fatty acids that have been associated with protection of the cell surface against environmental stress [46]. This protection is provided through both mechanical strengthening and enhanced chemical resistance to the apical membrane of cells. Sphingolipids have also been implicated in schistosomes in the formation of caveolae in the apical surface [47]. Regions of the adult *S. mansoni *tegument have been identified as detergent-insoluble glycosphingolipid-enriched membrane domains (DIG) and contain multiple caveolae structures [47]. We detected three potential homologs represented by two microarray probes, related to the Sphingolipid Metabolism Pathway (SMP) that were up regulated in adult males and females, lung schistosomula and eggs (Contig7899 lung schistosomula 2.5 fold, adult males 3.4 fold; Contig7987 eggs 3.1 fold, adult females 3.0 fold) of *S. japonicum *(Additional file 5). Glycosphingolipids have been identified as components of schistosome eggs [48] and are selectively bound by the antigen presenting cells of the liver by liver/lymph node specific ICAM-3-grabbing non-integrins [48]. A further role for sphingolipids in lung schistosomula has been previously suggested by El Ridi and Tallima [49] who demonstrated that an equilibrium of these lipids is critical for the selective absorption of small molecules across the lipid bilayer while also acting as a defence mechanism through the exclusion of antibody binding to the schistosome surface.

Six differentially expressed genes were identified as components of the Pentose Phosphate Pathway (PPP) (Additional file 6). These included genes encoding isomerases, gluconate dehydrogenases and proteins involved in fructose phosphorylation. The primary role of the PPP is anabolic rather than catabolic resulting in the production of nucleotides, RNA and DNA, and NADPH for reductive biosynthesis [50,51]. In addition, the PPP is an alternative to glycolysis for oxidation of glucose and energy production. Elevated expression of these genes was observed in eggs, miracidia, sporocysts, cercariae and adult female schistosomes, likely reflecting increased activity of this pathway (Contig6630 egg 7.9 fold, miracidium 2.7 fold, sporocyst 2.1 fold; Contig8868 egg 2.0 fold; TC7683 cercariae 2.1 fold; Contig6298 egg 2.4 fold; Contig8062 adult female 2.4 fold). Up-regulation of components of this pathway may reflect increased metabolism in the aquatic lifecycle stages where considerable tissue/cellular remodelling is required. Up-regulation of PPP components in the adult female of *S. japonicum *mirrors the extensive egg production and considerable cellular synthesis that are prominent biological features of this stage [52]. Surprisingly these PPP genes were not up regulated in the juvenile stages (lung schistosomula; liver stage/paired but immature male/female worms) which develop significantly in size and complexity. Further characterisation of PPP components as potential anti-schistosome drug targets may be rewarding, especially as the pathway has been targeted for drug development against trypanosomes and *Leishmania *[53,54]. Notably, one of these trypanosome drug targets is phosphogluconate dehydrogenase, a gene that was up-regulated in adult female *S. japonicum *(Contig8062; 2.4 fold).

Six genes of the Calcium Signalling Pathway (CSP) were up-regulated in the adult male of *S. japonicum*, as well as in the egg, miracidium, sporocyst and cercaria aquatic/snail stages (Contig7484 adult male 8.9 fold; Contig3653 cercaria 2.3 fold; Contig4586 adult male 3.1 fold; TC7426 egg 2.3 fold; Contig8713 egg 2.03 fold; Contig8558 egg 99.1 fold, miracidium 73.2 fold, sporocyst 4.0 fold) (Additional file 7). Elevated levels of sarco-endoplasmic reticulum ATPase (Contig4586 2.7–3.1 fold) in adult male worms probably reflect their requirements for increased muscle mass and the recognised importance of calcium in muscle physiology [55]. Of the other up-regulated CSP genes, those in the egg may be necessary for the translation of environmental signals to this stage, especially those required to initiate hatching following its exit from the mammalian host and its entry into fresh water. To expand on this, it is known that schistosome egg hatching requires appropriate regulation of calcium through calmodulin [56] which was highly upregulated in the egg and miracidium and also in the sporocyst of *S. japonicum *(Contig8558; egg, 71–99 fold; miracidium, 48–73 fold; sporocyst, 3–4 fold upregulated). Other genes within the CSP, especially those shown to be up-regulated within the egg of *S. japonicum*, may also encode components critical for hatching and/or other important biological functions and are worthy of further study.

## Conclusion

This report presents global gene expression patterns for the Asian blood fluke *S. japonicum *during its developmental cycle. Individual genes, gene ontologies and pathways that are modulated during the parasite lifecycle were also profiled. Using advanced filtering analyses including ANOVA, principal component analysis, and hierarchal clustering, we have provided a synopsis of relationships of gene expression among seven developmental stages of the schistosome. This represents the most comprehensive report to date on genes analysed for stage-specific expression in *S. japonicum*, indeed, in schistosomes in general, and should lead to a better understanding of development and differentiation in these parasites, their interaction with their mammalian and molluscan hosts, and how they modify gene regulation to adapt to a complex developmental cycle involving free-living and parasitic phases. The work may also provide new insights on schistosome-induced pathogenesis and has implications for developing new interventions for future schistosomiasis control. Indeed, the analyses revealed that gene expression profiles are linked to the major environmental settings through which the fluke progresses – from free-living aquatic miracidium, to parasitism of the snail by the sporocyst, to parasitism of the blood of the mammal by the maturing and adult schistosomes. As well as facilitating future research on gene regulation, at large, for a broad range of organisms with similar complex processes of development and differentiation, this resource should facilitate identification and prioritization of new intervention targets for the control and treatment of schistosomiasis.

## Methods

### Lifecycle maintenance and collection of *S. japonicum *life cycle stages

*Oncomelania hupensis hupensis *snails, infected with a Chinese mainland field isolate (from Anhui Province) of *S. japonicum*, were kindly provided by the National Institute of Parasitic Diseases, CDC, Shanghai. All samples of isolated parasite stages were resuspended in 1 ml of PBS and stored at -80°C until the total RNA was isolated. Viable schistosome eggs were obtained from infected mouse livers by digestion with collagenase B [57]. Eggs were used either for total RNA extraction or the production of miracidia. Miracidia were hatched and isolated as described [57]. Miracidia were either stored or used immediately for the production of sporocysts by incubation in MEMSE-j medium and maintenance in hypoxia chambers for 48 h under an atmosphere of 5%O_2_, 5% CO_2_, 90%N_2 _at 27°C [58,59]. Cercariae were obtained from surface water after being shed from infected snails exposed for 3 h to a bright light.

Lung schistosomula were isolated using modifications of a published procedure [60]. Approximately 1000 cercariae, pooled from several infected *Oncomelania *snails, were used to challenge female BALB/c mice. Three days later the lungs were removed, minced and incubated in RPMI at 37°C for 3 h on a rocker-shaker. The lung tissue solution was sieved and schistosomula were removed using a fine-tipped glass pipette. Adult worm pairs were perfused and separated by sex from female BALB/c mice challenged percutaneously with 30 cercariae of *S. japonicum *[7] at 4 (immature worms) and 6, 6.5 and 7 (mature, egg-producing worms) weeks post-infection.

### Total RNA isolation

Total RNA was isolated from parasites/free-living stages as described [61], with care being taken to ensure that all RNA samples were of high quality and quantity as assessed by Nanodrop ND-1000 spectrophotometer (A*260*/A*280 *nm ≥1.7 in nuclease-free water) and a Bioanalyzer [62].

### Microarray construction

The design and construction of the schistosome microarray [11] used in this study was facilitated by the availability of transcriptome data for *S. japonicum *[12] and *S. mansoni *[13] which added approximately 160,000 new schistosome ESTs (Expressed Sequence Tag) to GenBank. A large proportion of the EST sequences generated still require characterisation as only 45% of the *S. mansoni *and 65% of the *S. japonicum *ESTs showed similarity to sequences already in GenBank. Nevertheless, the two datasets likely represent the majority of the transcriptome of these two schistosome species [13]. The microarray comprises 19,222 target sequences printed twice from two independent probe designs, including 12,166 probes derived from *S. mansoni *contiguous sequences (contigs) and 7,056 probes derived from *S. japonicum *contigs. Variations to this initial design included the use of a second, independently formulated probe for each contig. Further details of the microarray design and the raw data from this study are presented in Supplementary Tables 1 and 2. Supplementary Information has been submitted at GEO-Gene Expression Omnibus,  accession numbers GPL7160, GSE12704.

### Microarray hybridisation and feature extraction

A 300 ng aliquot of total RNA from each developmental stage was used to synthesise fluorophore-labelled cRNA using Cyanine 3-CTP (CY3c) as described (One-Color Microarray-Based Gene Expression Analysis Protocol; Version 5.5, February 2007 Agilent). Samples were purified using the Qiagen RNeasy kit. Cyanine-labelled cRNA samples were examined at A*260 *and A*550 *using a ND-1000 spectrophotometer to determine yield, concentration, amplification efficiency and abundance of cyanine fluorophore. Once the concentration had been determined, 1.65 μg aliquots of CY3c were placed in a fresh tube together with the fragmentation mix (Agilent Technologies, Santa Clara, USA) and incubated for 30 min at 60°C. Then, the samples were combined with 2× Gene Expression hybridization Buffer HI-RPM, mixed and applied to a gasket slide that was pre-positioned in a hybridisation chamber (Agilent). The microarray slide was placed probe side towards the target. The chamber was assembled and placed in a hybridisation oven and incubated for 17 h at 65°C. After hybridisation the chamber was opened and the microarray slides were washed using the standard Agilent protocol (Agilent Technologies, USA) and scanned on an Agilent microarray scanner at 550 nm. Microarray hybridisations were performed in triplicate for all *S. japonicum *samples; additionally, biological replicates of adult male worms (collected at week 7) from separate infections were collected to determine any variation in gene expression. Microarray slides were scanned using an Agilent Microarray Scanner (B version). The "tag image format files" (tiff) produced by the scanner were loaded into the image analysis program Feature Extraction 9.5.3.1 (Agilent Technologies, USA) to establish standardised data for statistical analysis. All microarray slides were checked for background evenness by viewing the tiff image on Feature Extraction.

### Genespring analysis

Feature extracted data were analysed using GENESPRING software, version 7.3.1 (Agilent Technologies/Silicon Genetics, Redwood City, CA). Microarray data were normalised using the genespring normalisation scenario for "Agilent FE one-color" which including "Data Transformation: Set measurements less than 5.0 to 5.0", "Per Chip: Normalise to 50th percentile" and "Per Gene: Normalise to median".

Data sets were further analysed using published procedures [63] that consisted of methods related to one-colour experiments and utilised gProcessedSignal values determined using Agilent's Feature Extraction software including aspects of signal/noise ratio, spot morphology and homogeneity. ProcessedSignal represents signal after localised background subtraction and includes corrections for surface trends. Features were deemed *Absent *when the processed signal intensity was less than two fold the value of the processed signal error value. Features were deemed *Marginal *when the measured intensity was at a saturated value or if there was a substantial amount of variation in the signal intensity within the pixels of a particular feature. Features that were not *Absent *or *Marginal *were deemed *Present*. Data points were included only if *Present or Marginal *and probes were retained if all data points were *Present*. Analysis of Variance (ANOVA) was applied to this data using genes with statistically significant differences when grouped by lifecycle stage; parametric test, variances not assumed equal (Welch ANOVA); p-value cutoff 0.05; multiple testing correction: Benjamini and Hochberg False Discovery Rate [64]. About 5.0% of the identified genes would be expected to pass the restriction by chance. Principal Component Analysis (PCA) was run on conditions ie for each lifecycle stage, using mean centering and scaling.

### Protein blast and gene ontology analysis using Blast2Go

Batch BlastX (6 frame translation protein homology) was performed at  on al contigs. The Blast2go website was used to complement gene ontology and identify multiple genes within the Kyoto Encyclopedia of Genes and Genomes (KEGG) metabolic pathway maps . Gene ontology correlations with relative gene expression values were made using ErmineJ software [65]. Correlations for Biological Processes and Molecular Functions were made against microarray data using maximum gene dataset of 100, minimum dataset of 5 and maximum iterations of 10,000.

### Real time PCR

Gene expression patterns of a subset of genes were validated using real time PCR. Complementary DNA was synthesised from total RNA using a Qiagen QuantiTect whole transcriptome kit. Forward and reverse primers were designed from *S. japonicum *contigs. All cDNA samples synthesised from aliquots of the same total RNA used for the microarray hybridizations were diluted to 50 ng/μl, and quantified using a Nanodrop ND-1000 spectrophotometer. Subsequently aliquots of 1 μl were combined with 10 μl of SYBERs Green, 3 μl of water and 2 μl (5 pmol) of the forward and reverse primers in a 0.1 ml tube. All reactions were performed on a Rotor-Gene (3000) real time PCR and analyzed by Rotor Gene 6 Software. In order to minimise indiscriminate binding of double-stranded DNA, which can produce readings in the "no template" controls, separate reverse transcription and PCR steps were included. NADH-ubiquinone reductase was employed as a control (house-keeping) gene in the real time PCR studies [7]. Primer sets used are shown in Additional file 8.

## Availability

Supplementary Information has been submitted at GEO, accession numbers GPL7160, GSE12704. .

## Abbreviations

ANOVA: Analysis of variance; CSP: Calcium Signalling Pathway; DLC: dynein light chain; EST: Expressed Sequence Tag; GO: Gene Ontology; GEO: Gene Expression Omnibus; KEGG: Kyoto Encyclopedia of Genes and Genomes; PCA: Principal Component Analysis; PPP: Pentose Phosphate Pathway; SAGE: serial analysis of gene expression; Sj: *Schistosoma japonicum*; Sm: *Schistosoma mansoni*, SMP: Sphingolipid Metabolism Pathway.

## Authors' contributions

GG designed the study, analysed data and wrote the manuscript. DM and PB designed the study and wrote the manuscript. LM performed the microarray and real time PCR experiments.

## Supplementary Material

Additional file 1 **Filtering of data from triplicate hybridisation of 38,444 probes to schistosome transcripts representing 19,222 genes.** After filtering for "flagged" genes against all hybridisations, 7,132 probes were left, representing 4,371 genes; a final ANOVA of this dataset retained 6,465 probes and 4,104 genes. E, eggs; M, miracidia; S, sporocysts; C, cercariae; L, lung schistosomula; F4, juvenile females; M4, juvenile males; F6, F6.5, F7, adult female worms analysed at 6, 6.5 and 7 weeks post-cercarial challenge; M6, M6.5, M7, adult male worms analysed at 6, 6.5 and 7 weeks post-cercarial challenge. Click here for file

Additional file 2 **Complete list of differentially expressed *S. japonicum *genes after filtering for flags.** Genes enriched 2 fold or higher (sorted in decreasing order of expression) for each of the lifecycle stages are shown on different sheets for the egg, Miracidium, Sporocyst, Cercaria, Lung Schistosomulum, Juvenile Female (F4), Juvenile Male (M4), Adult Female (combined weeks 6–7), Adult Male (combined weeks 6–7). Click here for file

Additional file 3 **Complete overview of the custom designed schistosome microarray manufactured by Agilent Technologies used in this study.** Click here for file

Additional file 4 **Comparison of findings of Jolly et al.**[9] examining *S. mansoni *and the current study examining *S. japonicum*. Click here for file

Additional file 5 **KEGG of sphingolipid metabolism. *S. japonicum *genes that were upregulated in the life cycle stages shown are highlighted in red.** Click here for file

Additional file 6 **KEGG of the pentose phosphate pathway. *S. japonicum *genes that were upregulated in the life cycle stages shown are highlighted in red.** Click here for file

Additional file 7 **KEGG of the calcium signalling pathway. *S. japonicum *genes that were upregulated in the life cycle stages shown are highlighted in red.** Click here for file

Additional file 8 **Primer Sets for real time PCR validation of a subset of genes that were upregulated in each of the lifecycle stages examined.** Click here for file
