# Supplementary figures and images for: Developmental gene expression profiles of the human pathogen Schistosoma japonicum
Source: BMC Genomics. 2009 Mar 25;10:128. doi: 10.1186/1471-2164-10-128 (PMC2670322; doi:10.1186/1471-2164-10-128)

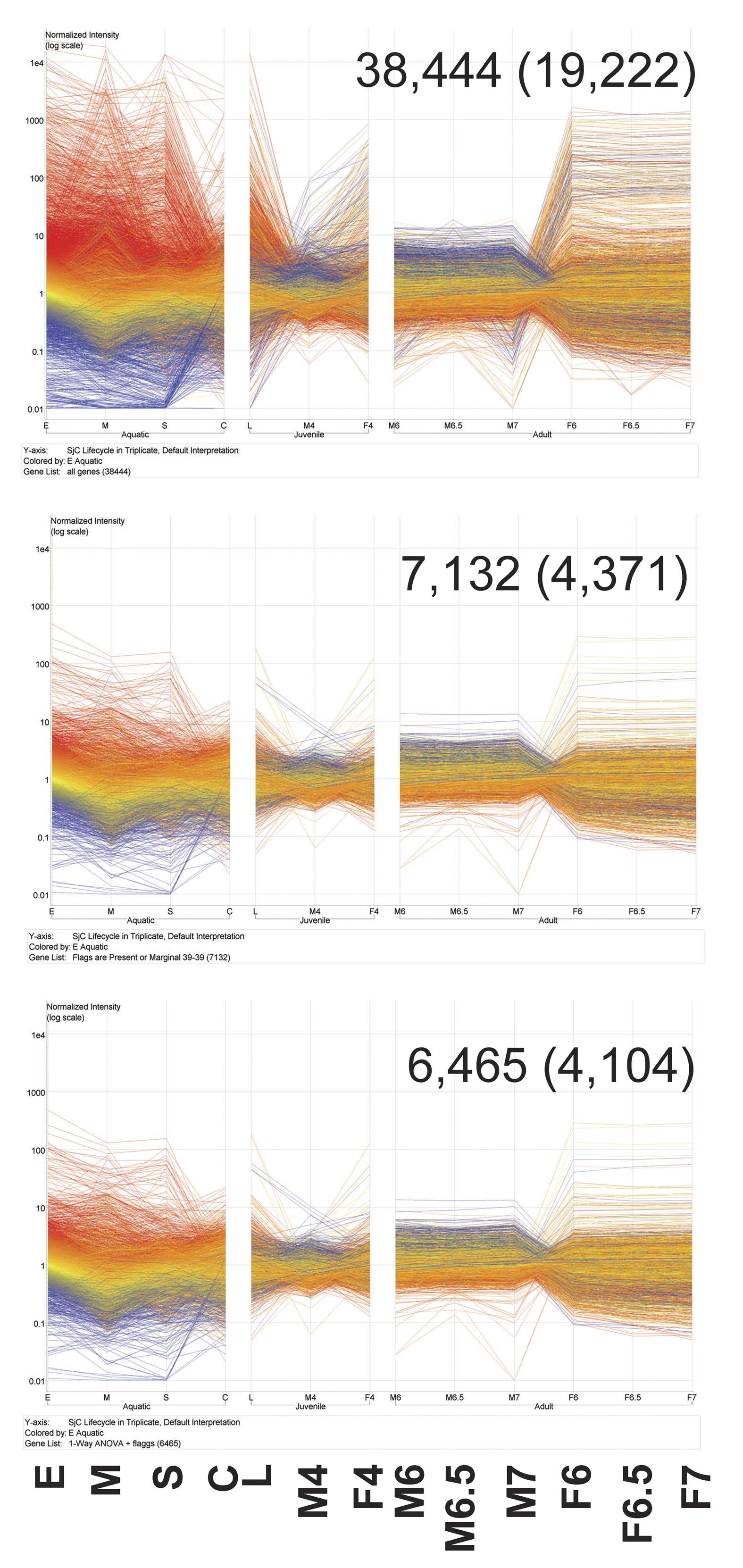

Supplement: Additional file 1 — Filtering of data from triplicate hybridisation of 38,444 probes to schistosome transcripts representing 19,222 genes. After filtering for "flagged" genes against all hybridisations, 7,132 probes were left, representing 4,371 genes; a final ANOVA of this dataset retained 6,465 probes and 4,104 genes. E, eggs; M, miracidia; S, sporocysts; C, cercariae; L, lung schistosomula; F4, juvenile females; M4, juvenile males; F6, F6.5, F7, adult female worms analysed at 6, 6.5 and 7 weeks post-cercarial challenge; M6, M6.5, M7, adult male worms analysed at 6, 6.5 and 7 weeks post-cercarial challenge. [file 1471-2164-10-128-S1.jpeg]

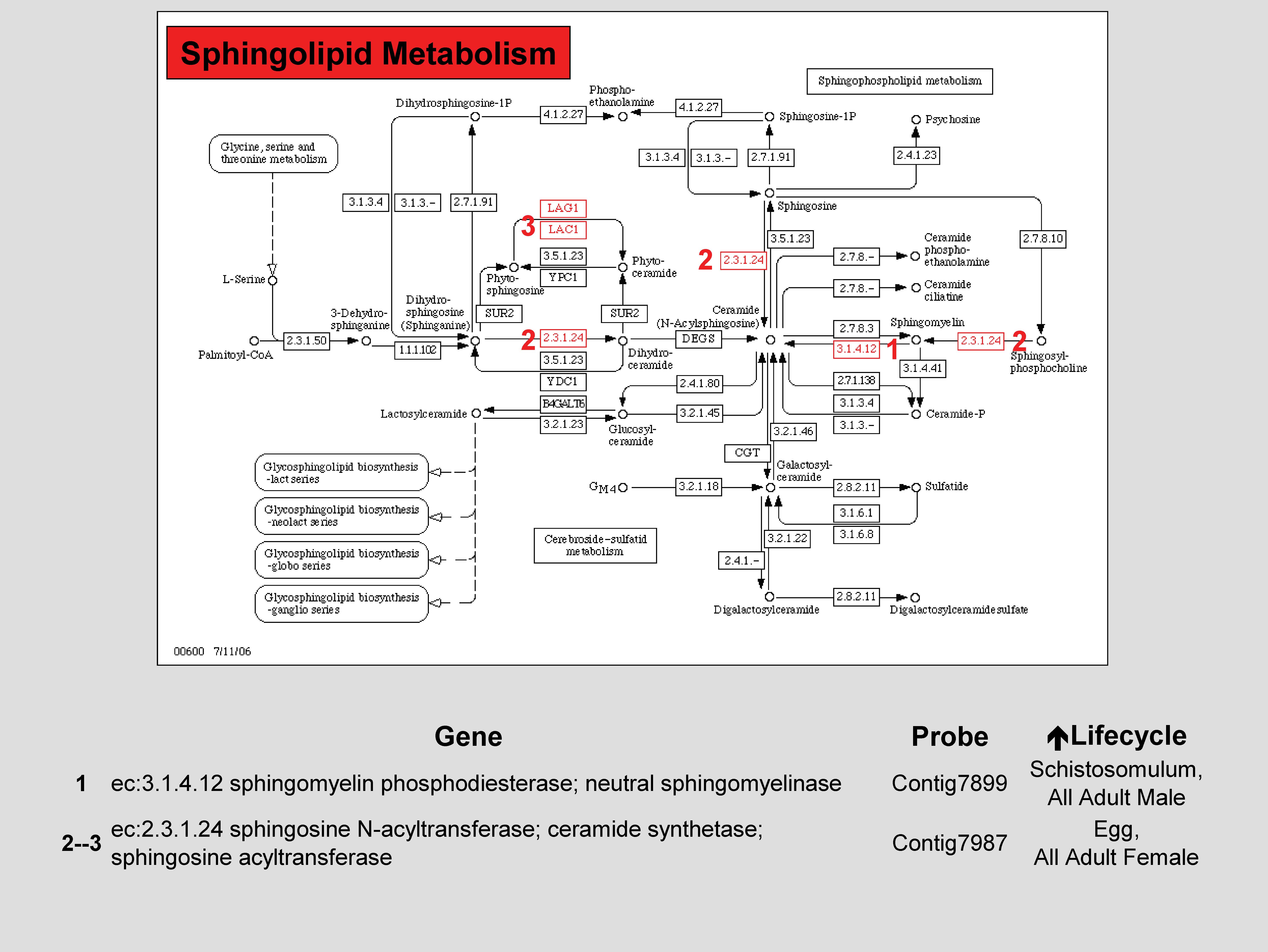

Supplement: Additional file 5 — KEGG of sphingolipid metabolism. S. japonicum genes that were upregulated in the life cycle stages shown are highlighted in red. [file 1471-2164-10-128-S5.jpeg]

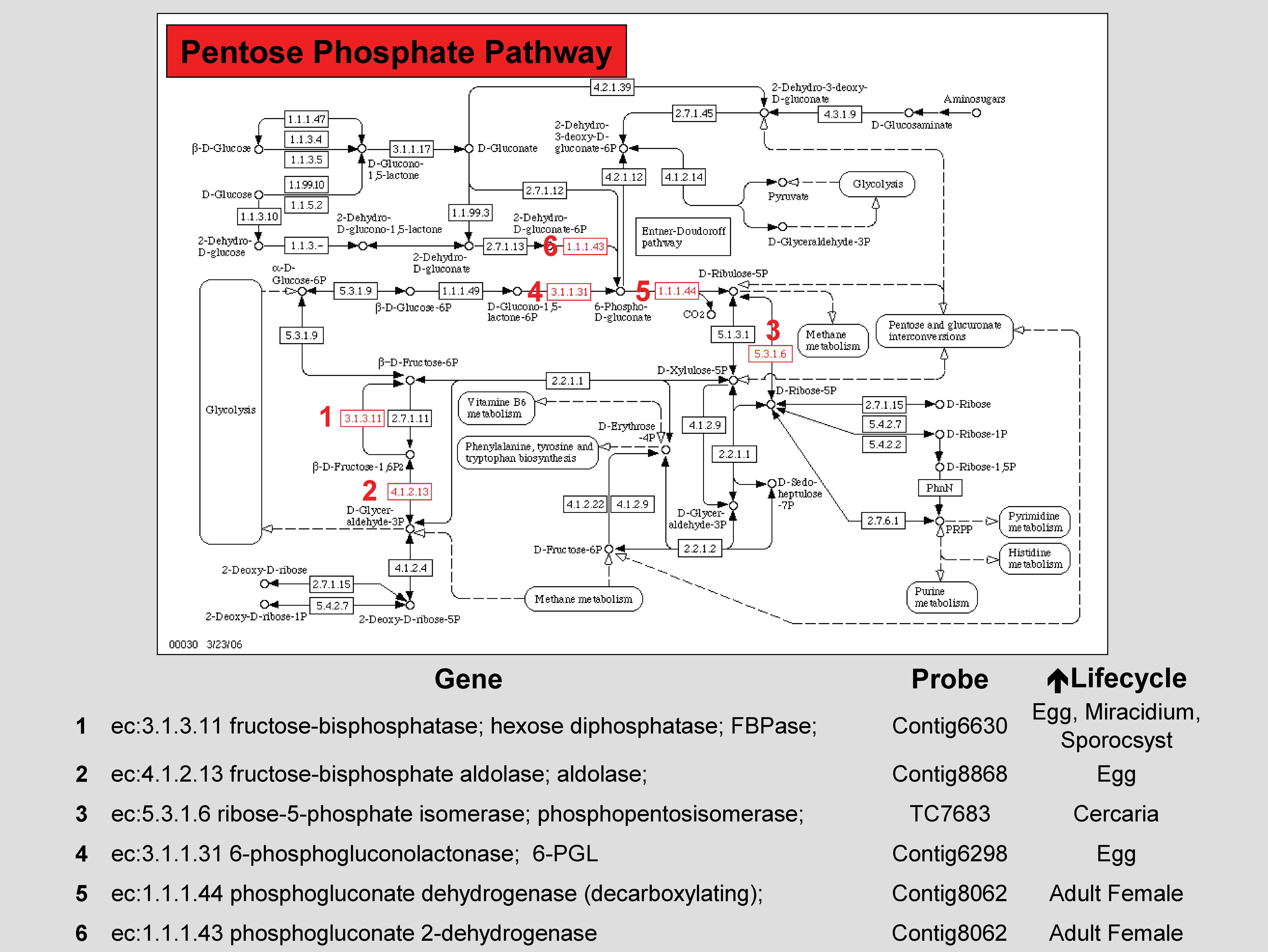

Supplement: Additional file 6 — KEGG of the pentose phosphate pathway. S. japonicum genes that were upregulated in the life cycle stages shown are highlighted in red. [file 1471-2164-10-128-S6.jpeg]

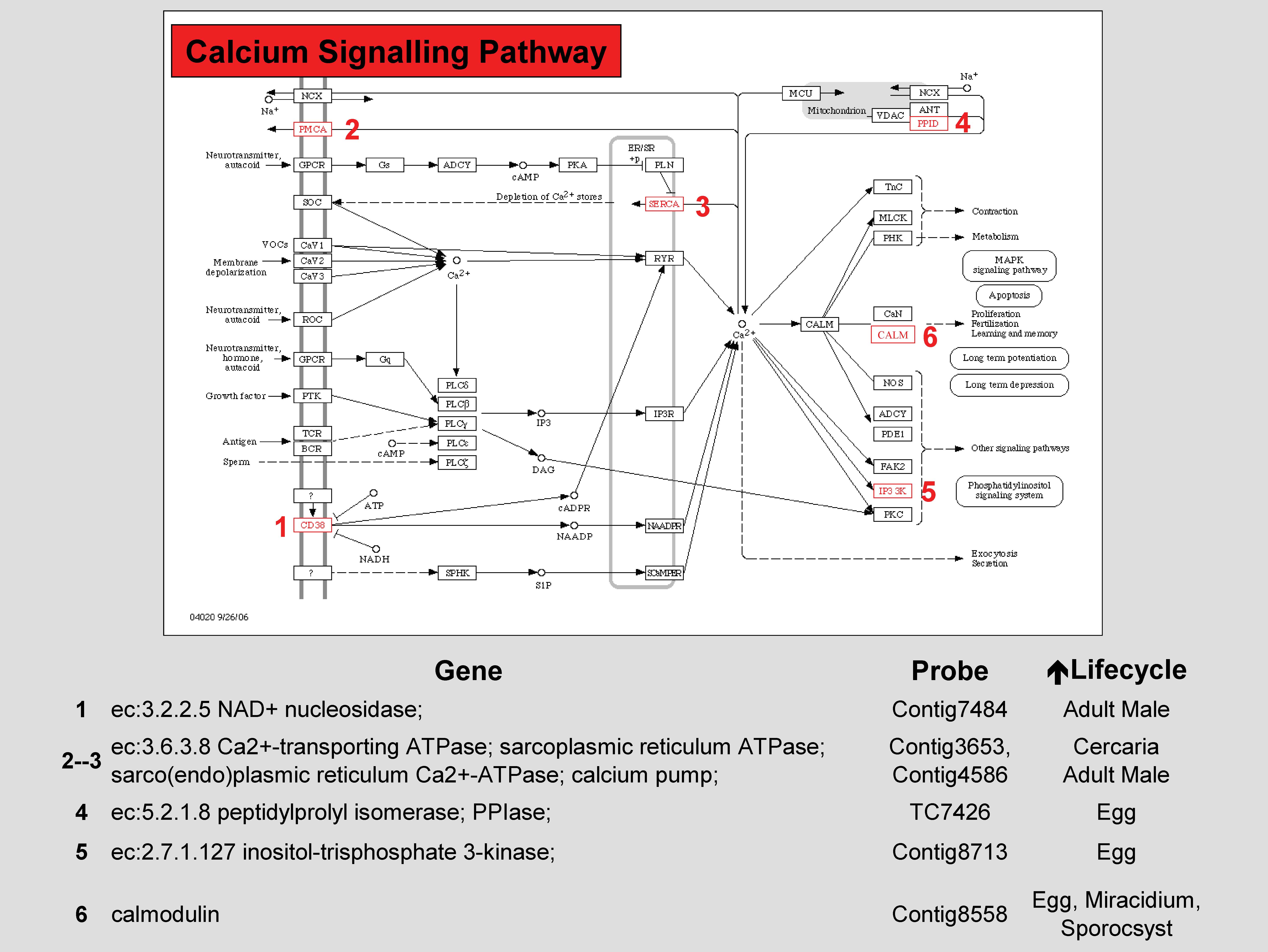

Supplement: Additional file 7 — KEGG of the calcium signalling pathway. S. japonicum genes that were upregulated in the life cycle stages shown are highlighted in red. [file 1471-2164-10-128-S7.jpeg]
